# Pathogenesis of atherosclerosis in the tunica intima, media, and adventitia of coronary arteries: An updated review

**DOI:** 10.17305/bjbms.2019.4320

**Published:** 2020-02

**Authors:** Aleksandra Milutinović, Dušan Šuput, Ruda Zorc-Pleskovič

**Affiliations:** 1 Institute of Histology and Embryology, Faculty of Medicine, University of Ljubljana, Ljubljana, Slovenia; 2 Institute of Pathophysiology, Medical Faculty, University of Ljubljana, Ljubljana, Slovenia; 3 International Center for Cardiovascular Diseases MC Medicor d.d., Izola, Slovenia

**Keywords:** Atherosclerosis, intima, media, adventitia, perivascular adipose tissue

## Abstract

Atherosclerosis is a chronic inflammatory disease of arteries and it affects the structure and function of all three layers of the coronary artery wall. Current theories suggest that the dysfunction of endothelial cells is one of the initial steps in the development of atherosclerosis. The view that the tunica intima normally consists of a single layer of endothelial cells attached to the subendothelial layer and internal elastic membrane has been questioned in recent years. The structure of intima changes with age and it becomes multilayered due to migration of smooth muscle cells from the media to intima. At this stage, the migration and proliferation of smooth muscle cells do not cause pathological changes in the intima. The multilayering of intima is classically considered to be an important stage in the development of atherosclerosis, but in fact atherosclerotic plaques develop only focally due to the interplay of various processes that involve the resident and invading inflammatory cells. The tunica media consists of multiple layers of smooth muscle cells that produce the extracellular matrix, and this layer normally does not contain microvessels. During the development of atherosclerosis, the microvessels from the tunica adventitia or from the lumen may penetrate thickened media to provide nutrition and oxygenation. According to some theories, the endothelial dysfunction of these nutritive vessels may significantly contribute to the atherosclerosis of coronary arteries. The adventitia contains fibroblasts, progenitor cells, immune cells, microvessels, and adrenergic nerves. The degree of inflammatory cell infiltration into the adventitia, which can lead to the formation of tertiary lymphoid organs, correlates with the severity of atherosclerotic plaques. Coronary arteries are surrounded by perivascular adipose tissue that also participates in the atherosclerotic process.

## INTRODUCTION

Atherosclerosis is a chronic inflammatory disease of arteries. Despite extensive research on atherosclerosis in the past years, the complex pathogenesis of disease is still not completely clear. Nevertheless, new techniques and methods provide new clues about the development of atherosclerosis and challenge the traditional assumptions.

The coronary arteries (CAs) supply the heart with blood. They originate at the base of the aorta from openings located just behind the aortic valve leaflets. The wall of CAs consists of three layers: the innermost layer (tunica intima), the middle layer (tunica media), and the outermost layer (tunica adventitia or externa); all encapsulated in perivascular adipose tissue (PVAT) [1,2].

Atherosclerotic changes in the vessel wall characterize coronary artery disease (CAD). Atherosclerosis affects the structure and function of all three layers of the CA wall [1,3].

### The tunica intima of CAs

The intima consists of a lining layer of longitudinally oriented endothelial cells that cover the subendothelial layer of thin connective tissue with sparsely cellular matrix and the internal elastic membrane (lamina) [4,5]. The endothelial cells secrete numerous bioactive substances [3] to modulate vascular tone, prevent the platelet and leukocyte adhesion, control the process of thrombosis, prevent the invasion of harmful substances into the arterial wall, and act as a selective diffusion barrier between the blood and the other wall layers [3,6]. The intima is separated from the media by the internal elastic membrane, which is composed of fenestrated layer of elastic fibers. With advancing age or intimal disease, the internal elastic membrane may be fragmented, duplicated, or focally lost [6].

**FIGURE 1:**
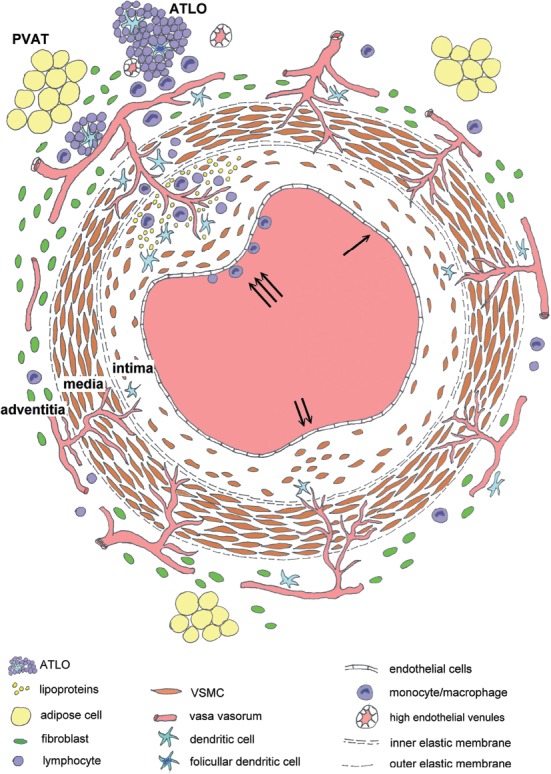
A schematically illustrated mechanism of atherosclerosis in CAs [7]: ↑ diffuse intimal thickening; ↑↑ pre-pathological intimal thickening with cell proliferation and production of a higher amount of modified extracellular matrix; ↑↑↑ pathological intimal thickening and its progression to fibroatheroma with neovascularization, deposition of low density lipoproteins covered with a layer of VSMCs in the outer part of intima, and followed by inflammatory infiltration. CA: Coronary artery; VSMC: Vascular smooth muscle cell; ATLO: Adventitial tertiary lymphatic organ.

The intima of a newborn is composed of a single-cell layer, and with age, it gets thicker and becomes multilayered [7], depending on the location along the heart-periphery axis and on the hemodynamics of blood flow [8-10]. In adults, the intima of CAs is thicker than the media [7,11]. This diffuse intimal thickening (Figure 1) is considered to be [4,5,7] a normal [12] or benign process [13-15], because it does not necessarily progress to atherosclerosis [16].

The subendothelial layer formed in the intima consists of vascular smooth muscle cells (VSMCs) and the extracellular matrix (ECM) that is rich in longitudinally oriented elastic fibers and proteoglycans [5]. Dendritic cells are also present [17,18]. VSMCs are longitudinally oriented and arranged in several layers. They have a stable phenotype and a very low proliferative rate [5,19,20]. The density of cell layers and elastic fibers increases toward the media [7]. Focally, in response to harmful proatherogenic stimuli, VSMCs start to proliferate and they produce a high amount of modified ECM, forming pre-pathological intimal thickening [pre-PIT] (Figure 1) [4,5].

In the early stage of atherosclerosis, called PIT (Figure 1), lipids accumulate in the ECM, in deeper regions of the subendothelial layer. They form lipid pools, rich in proteoglycans and hyaluronan, covered with a layer of VSMCs [7,16,21]. In this part of the subendothelial layer, toward the media, the ECM proteoglycan biglycan facilitates binding, retention, and deposition of low-density lipoprotein cholesterol (LDL-C). At this stage, the inner part of the subendothelial layer, just below endothelial cells, is still poor in VSMCs and biglycan and is free from lipoprotein deposition [4,7]. Current theories suggest that endothelial injury is one of the initial steps in the development of atherosclerosis. It may occur in the endothelium surrounding the lumen of the mother vessel or in the endothelium of vasa vasorum, or both. The response-to-retention and the response-to-injury are two major theories that support the idea of dysfunctional endothelium that surrounds the lumen of a large vessel. In the response-to-injury hypothesis, an initial injury (mechanical injury or toxins) leads to endothelial dysfunction and the passage of inflammatory cells, especially macrophages and T cells, into the arterial wall, followed by the proliferation of VSMCs [22,23]. The response-to-retention hypothesis states that, in response to predisposing stimuli (mechanical strain and cytokines), the initial event is the retention of lipoproteins bound to the ECM in the intima [4,24,25]. Lipoproteins enter the arterial wall through dysfunctional endothelium that surrounds the lumen of the vessel, and this process is followed by the entry of monocytes and other inflammatory cells [5].

According to the response-(to-neovascularization)-to-retention hypothesis, the initial events of coronary atherosclerosis are enlarged intimal thickness, cell hypoxia, and intimal neovascularization by vasa vasorum, followed by lipoprotein accumulation [7]. Vasa vasorum are small blood vessels responsible for the nutrition of the walls of large vessels. Vasa vasorum are not detectable in the arteries of infants, not even in the ascending aorta. With the growth of a child the thickness of vessel walls in the arterial system increases, because of an increase in wall tension. The diffusion through the thick wall thus becomes increasingly limited, due to increased diffusion distance [26]. The cells in the outer part of thickened intima become hypoxic, which leads to neovascularization from the adventitial part - as vasa vasorum externa [7,26,27] or from the luminal part - as vasa vasorum interna [1,28]. The newly formed vessels have endothelial cells highly permeable for lipoproteins and inflammatory cells, which enter and accumulate in deeper intima [7,27].

The response-to-injury of vasa vasorum hypothesis suggests that the leading cause of atherosclerosis is specifically the injury of vasa vasorum [26]. According to this hypothesis, the initial event of atherosclerosis is disruption or occlusion of vasa vasorum leading to ischemia of the mother vessel wall [26]. This hypothesis proposes that atherosclerosis is a microvascular disease rather than a large-vessel disease [26]. Because vasa vasorum are functional end arteries, their obstruction results in ischemic necrosis of the cells in the subintimal layers in the areas supplied by vasa vasorum. This could be a possible cause of focal distribution of atherosclerotic lesions. Areas predictably spared from atherosclerosis are intramyocardial bridges and mammary arteries. They have a few if any vasa vasorum and thus cannot suffer from vessel wall ischemia from disturbed microcirculation [26]. Many risk factors [29,30], such as hypertension, stress, smoking, and increased PVAT in obesity are more effective for endothelial damage in small blood vessels than in large ones. Endothelium dysfunction and the subsequent thrombotic events would cause more damage in microvessels than in larger arteries. Obstruction of vasa vasorum further leads to functional impairment and structural damage of the large mother vessel [26]. It was shown that physical constriction of vasa vasorum precipitates fatty streak development in the underlying arterial segment, and obstruction of vasa vasorum in the abdominal aorta results in the formation of an aneurysm [31].

The atherosclerotic lesions are mostly located in atheroprone regions. These regions are predominantly found at the branches and curvatures of large vessels. The blood flow in branches and curvatures is disturbed with nonuniform and irregular low wall shear stress, which is detected by endothelial primary cilia sensitive to the shear stress [32,33]. This disturbed flow and low shear stress modulate the expression and structure of permeability-related intercellular junctional proteins in endothelial cells [34]. The endothelial barrier becomes permeable for lipoproteins and this leads to the accumulation and sequestration of LDLs in the intima [35]. These lipoproteins in the intima are then modified by numerous mechanisms, such as oxidation by reactive oxygen species (ROS) and enzymatic cleavage, which transforms them into proinflammatory lipoproteins [36]. Endothelial cells secrete increased amounts of vasoconstricting factors (mainly endothelin-1) and decreased amounts of vasodilating factors (especially nitric oxide). They also express molecules that promote and amplify the adherence and migration of leukocytes, mainly monocytes, to the vessel wall, such as vascular cell adhesion molecule (VCAM), intracellular adhesion molecule (ICAM), and monocyte chemotactic protein 1 [MCP-1] (Figure 1) [3,23].

In the intima, monocytes acquire characteristics of proinflammatory macrophages, which express the scavenger receptors and internalize modified lipoproteins. The infiltration and proliferation of macrophages is a characteristic feature of the progression of PIT to fibroatheroma stage (Figure 1). Macrophages give rise to local inflammation by secreting various inflammatory cytokines (e.g., tumor necrosis factor alpha [TNFα], interleukin-1 [IL-1], and IL-6), which recruit T cells, B cells [37], and more macrophages into the lesion site [38]. Furthermore, VSMCs differentiate into macrophage-like cells [39] and together with the infiltrated macrophages extensively phagocyte modified and oxidized LDLs, hyaluronan, and versican [16]. Lysosomal malfunction in macrophages and macrophage-like cells leads to the accumulation of oxidized lipoproteins and cholesterol and the transformation into foam cells. Foam cells undergo apoptosis and accumulate calcium in acidic extracellular space. The increased apoptosis, decreased uptake of apoptotic bodies, and altered composition of ground substance with a decreased amount of versican, biglycan, and hyaluronan are characteristic features of necrotic core formation in fibroatheroma [40-42]. A thick fibrous cap containing VSMCs surrounds the necrotic core and stabilizes the vulnerable plaque [43]. Macrophages, T cells, and B cells as well as dendritic cells, frequently present in this region in high numbers [18,44], produce cytokines (i.e., TNFα, IL-1β, and IL-6) that induce apoptosis of VSMCs or differentiation of VSMCs into cells with osteochondrogenic phenotype, which promote mineral deposition in the atherosclerotic plaque [43,45,46].

The next step in atherosclerosis progression is the formation of thin-cap fibroatheroma. This structure has a thin fibrous cap, infiltrated with macrophages and T cells, and a large necrotic core. These advanced atherosclerotic plaques also show intraplaque neovascularization, where the extravasation of platelets and red blood cells into plaques contributes to the formation and expansion of the necrotic core. Thin-cap fibroatheroma is prone to rupture and thrombosis. After the erosion or rupture of the plaque, injured endothelial cells excrete increased amounts of thrombotic factors (e.g., von Willebrand factor [VWF] and thromboxane A2 [TXA2]) and decreased amounts of antithrombotic factors (e.g., heparin) leading to atherothrombosis [3,47-49].

In atherosclerotic lesions, various cytokines and modified lipoproteins influence the phenotype of macrophages, affecting the course of atherosclerotic lesions [35,39]. Proinflammatory and proatherogenic M1 macrophages are found in rupture-prone lesions and are associated with plaque vulnerability. M4 macrophages also display proinflammatory profile but low phagocytic activity. M4 macrophages are found in coronary atherosclerotic lesions, where they were associated with plaque instability [50]. Hemoglobin/haptoglobin complexes enter M2 macrophages through CD163 receptors leading to a specific atheroprotective type of macrophages, i.e., M(hem) or M(Hb) macrophages [51]. They can remove iron and prevent foam cell formation. M2 macrophages display high oxidative capacity but M(hem) and M(Hb) macrophages have reduced oxidative capacity [35,39].

### Besides macrophages, different subsets of T cells are found in atherosclerotic lesions.

They exhibit either proinflammatory (proatherogenic) or antiinflammatory and immunoregulatory (antiatherogenic) properties. T helper (Th) cells are known to modulate the formation of atherosclerotic lesions. It was shown that Th1, Th9, Th22, and natural killer (NK) cells have a proatherogenic function, while Th2, Th17, and T regulatory (Treg) cells have an atheroprotective function in the development of atherosclerosis [18,49]. Proatherogenic T cytotoxic cells are less frequent than Th cells in the early atherosclerotic lesion. However, in advanced human lesions, for instance in rupture-prone plaques, they can count up to the half of a T cell population [49,52-54]. In addition, low amounts of B cells and, most likely, B2-derived plasmablasts are found only in advanced plaques in humans [18,49,55]. In the neointima of CAs with cardiac allograft vasculopathy the most numerous were Th1 cells, while macrophages and B cells were in small numbers [56].

### The tunica media of CAs

The media of CAs consists of multiple layers of VSMCs (Figure 1) and the ECM with circular elastic fibers, collagen, and proteoglycans [1,2,6]. CAs are muscular arteries, which means that they have a thicker media with more VSMCs, compared to elastic arteries [2]. VSMCs in the media are oriented circumferentially or helically in up to 40 layers. The media of normal CAs ranges in thickness from 125 to 350 µm (average 200 µm) [6]. The media underlying intimal atherosclerotic plaque is considerably thinner, ranging from 16 to 190 µm (average 80 µm) [6]. The external elastic membrane, which separates the media from the adventitia, is thinner than the internal elastic membrane. It is composed of interrupted layers of elastin. Neurotransmitters released from the unmyelinated axons that are closely adherent to the outer border of the external elastic membrane diffuse through the fenestrations of the external elastic membrane and stimulate medial VSMCs. Propagation of depolarization of VSMCs occurs in the media through low-resistance gap (nexus) junctions [57].

Except in special circumstances, VSMCs are the only cells in the media and have both contractile and synthetic functions [1,58]. They are usually of contractile phenotype and express various contractile proteins such as smooth muscle α–actin (α-SMA or ACTA2), SM-22α, SM myosin heavy chains SM-1 and SM -2, calponin, and smoothelin [43]. VSMCs respond to signals such as acetylcholine and norepinephrine; they regulate vessel diameter and blood flow and maintain arterial tone through contraction and relaxation in opposition to the heart [1,43,59]. VSMCs also maintain the ECM of media. These cells synthesize short-lived collagen fibers, long-lived elastic fibers, and the constituents of ground substance [1]. In normal circumstances, VSMCs have a low proliferative rate.

VSMCs are not terminally differentiated and display phenotypic plasticity [60]. In response to injuries, such as tissue damage or other local stimuli, VSMCs can transitionally alter their phenotype from contractile to synthetic state, which includes downregulating contractile proteins, increasing proliferation, and remodeling the ECM to facilitate migration. After the repair has been completed, VSMCs return to contractile state [43].

During atherogenesis, VSMCs also undergo phenotypic changes and selectively proliferate to and accumulate in the vessel wall [61]. They lose the expression of α-SMA and express markers of mesenchymal stem cells (MSCs) and myofibroblasts [62,63].

In response to a variety of stimuli, VSMCs can also display markers and features of osteoblasts, chondrocytes, adipocytes, and macrophage-derived foam cells. The transformation from the normal contractile type of VSMCs into osteogenic/chondrogenic phenotype leads to calcification of the vessel wall [64]. In advanced lesions of CAs, 40% of foam cells expressed both the VSMC marker ACTA2 and the macrophage marker CD68. It was not clear, however, whether the foam cells were macrophage- or VSMC-derived cells [43]. Variety of other cell types, such as multipotential vascular stem cells, adipose cells, fibroblasts, and macrophages can differentiate and gain VSMC marker expression [65].

At birth, the intima is thin and the media is avascular [1]. With aging, coronary intima thickens (0.35–0.5 mm) and the microvessels from the adventitia or the lumen penetrate media (Figure 1) [1,7,26-28]. At this stage, in addition to VSMCs, endothelial cells are present in the media [1]. Dendritic cells, which are found in the intima along the subendothelial layer and in the adventitia close to vasa vasorum, have not been identified in normal media (Figure 1) [17,18]. Rare individual resident or infiltrated leukocytes may also be present in the media of normal CA wall. However, in CAs of young people dying of trauma resident leukocytes were not found in the media [1].

The media is immunoprivileged – it develops active and passive protective mechanisms against inflammation. Passive mechanisms include the presence of elastic membranes and the rarity or absence of blood and lymphatic vessels [66]. In active immune privilege, VSMCs play a crucial role. Inflammatory cells, such as macrophages and T cells, are infiltrated primarily into the intima and adventitia of atherosclerotic vessel wall. The inner and outer elastic membranes, which function as a barrier to leukocyte trafficking, prevent the infiltration into the media (Figure 1). The cytokine interferon gamma (IFNγ), secreted by inflammatory cells in the intima and adventitia, diffuses into the media and activates VSMCs to synthesize indoleamine 2,3-dioxygenase 1 (IDO1) [67,68] and transforming growth factor beta (TGFβ) [69]; in addition, VSMCs express low amounts of major histocompatibility complex (MHC) class II molecules [70]. Besides VSMCs, macrophages [71,72] and endothelial cells secrete IDO1, but of much less sensitivity [1]. IDO1 is an enzyme that catalyzes degradation of tryptophan. Tryptophan degradation or kynurenine metabolite production in the microenvironment of media modulates T cell function [73] by preventing clonal proliferation, promoting apoptosis, and generating atheroprotective regulatory T cells [1].

Early in the immune response, infiltrated innate leukocytes can stimulate VSMCs and endothelial cells to produce proinflammatory cytokines, including IL-1, IL-6, and TNFα [74], which recruit additional leukocytes from the circulation [1]. The disruption of media immune privilege manifests as the damage and loss of VSMCs, destruction of the ECM architecture, and more intense leukocyte infiltration [1,2]. However, in the media of CAs with advanced confluent stable plaques [2] and in CAs with cardiac allograft vasculopathy [56] 5- to 10-fold fewer inflammatory cells were detected than in the intima or adventitia. In both studies, T cells were the most numerous, followed by macrophages and B cells [2,56]. The inflammatory infiltration of the media of CAs was also found in patients with ischemic cardiomyopathy who underwent cell transplantation and in patients with fatal myocardial infarction [75]. The primary cell type were T cells, followed by B cells and some dendritic cells [75].

### The tunica adventitia and PVAT of CAs

The adventitia consists of connective tissue with collagen and elastic fibers, vasa vasorum, adrenergic nerves, and lymphatic vessels. The thickness of the adventitia in CAs ranges from 300 to 500 µm [6]. Longitudinally oriented collagen bundles primarily permit continual changes in CA diameter [57].

Fibroblasts are the main cells in adventitial connective tissue (Figure 1) [3,76]. The adventitia also contains progenitor cells [77,78] and immune cells such as macrophages [79,80], T cells [81], B cells [79,80], and dendritic cells [79,80,82]. In response to injury and stress, fibroblasts from the adventitia proliferate, differentiate into myofibroblasts, and migrate to the intima [3,83]. They secrete factors that regulate the growth of endothelial cells [83] and VSMCs and recruit inflammatory and progenitor cells to the vessel wall [3,84].

The CAs are surrounded by PVAT (Figure 1) [85]. The adipocytes in PVAT are not terminally differentiated cells. They are smaller in size, accumulate fewer cytoplasmic lipid droplets, and have a more irregular shape compared to subcutaneous or perirenal adipocytes, but their phenotype is more similar to white than to brown adipocytes [86]. It was shown that, in response to cold stimuli, PVAT has thermogenic properties similar to brown adipose tissue. In cold environment, PVAT releases prostacyclin that improves endothelial function and inhibits atherosclerosis in mice [87]. Other studies showed that human PVAT releases adiponectin, which possesses antiinflammatory, insulin-sensitizing [88], and vasodilating properties [85,89-93].

Other PVAT-derived vasoactive factors can attenuate endothelial-dependent relaxation or increase vascular contractility [85]. PVAT protects CAs against wave torsion [85,94]. Genes overexpressed in PVAT are involved in ECM remodeling, inflammation, infection, thrombosis, cell cycle, O-N glycan biosynthesis, and sphingolipid metabolism. Sphingolipids induce aggregation and retention of oxidized lipoproteins in the subendothelial space [94], which is associated with the development and progression of atherosclerosis [85,94].

Following the discovery of the correlation between the degree of inflammatory infiltration in the adventitia and the severity of atherosclerotic plaques in CAs, numerous studies have investigated the components of adventitial cell aggregations, in humans and animal models [75,79,80,95-98]. These cell aggregations, which can be more or less well-organized structures, are known as tertiary lymphatic organs (TLOs) [75]. Adventitial TLOs (ATLOs) are well-organized structures with areas of T cells, B cells, antigen-presenting cells such as follicular dendritic cells, lymphatic vessels, and high endothelial venules (Figure 1) [75,99-101]. Recently, key genes and pathways involving cytokine-cytokine receptor interaction and chemokine signaling have been associated with the formation of ATLOs in atherosclerosis in mice [102].

In the abdominal aorta of Apoe−/− mice, ATLOs were associated with atherosclerotic lesions in the intima, which indicated that a crosstalk between the intimal lesions and ATLOs occurs via the media [75,103]. The following classification of ATLO stages in mice was developed: stage I - ATLO mostly contains aggregates of T cells; stage II - ATLO with mixed T and B cell aggregates where T/B cells are in separate areas; and stage III - ATLO with ectopic germinal centers and separate T and B cell areas [75]. Furthermore, Akhavanpoor et al. applied the murine stage classification on 72 CAs from patients that suffered either from dilated cardiomyopathy, ischemic cardiomyopathy, or fatal myocardial infarction, as follows: stage I - low number of cells, T cell areas; stage II -separate T cell and B cell areas in mixed aggregates; and stage III - follicular dendritic cell networks, separate T cell and B cell areas. The stage of ATLO was in correlation with plaque size and with the instability and rupture of plaque. Stage III was found in all patients with myocardial infarction [75]. Patients with dilated cardiomyopathy had no ATLOs in the adventitia. Patients with ischemic cardiomyopathy had ATLOs with the most numerous B cells (54%), followed by T cells (31%), dendritic cells (3%) and unknown [rest] (12%) cells [75]. Patients with myocardial infarction had ATLOs with B cells (38%), T cells (33%), dendritic cells (4%), and unknown [rest] cells (25%) [75].

Many studies investigated the role of different cell types, associated with inflammation in the adventitia, in the development of atherosclerosis. Th1 cells are proatherogenic and secrete proinflammatory cytokines such as IL-2, TNFα, and IFNγ. NK cells also display proatherogenic function. B2 cells play a proatherogenic role to stimulate Th1 cells and dendritic cells and to secrete immunoglobulin G (IgG). Proatherogenic cells are also innate response activator (IRA) B cells, which release stimulating factors for dendritic cells. Th2 cells and Th17 cells have both proatherogenic and atheroprotective function, while Treg cells and B1 cells play an atheroprotective role. Treg cells release antiinflammatory cytokines (e.g., IL-10, IL-35, and TNFβ) and B cells secrete IgM [3,18].

## CONCLUSION

Endothelial cell damage is an important early stage in the development of atherosclerosis. Migration of VSMCs to the intima is a prerequisite for the advancement of the disease. However, previous research also suggests that a multilayered intima consisting of VSMCs is normal adaptation of CAs to rheological conditions rather than pathology on its own. The onset of atherosclerosis is endothelial dysfunction followed by other pathological processes, such as the transformation of cells in the vascular wall that leads to atherosclerosis. The thickening of the arterial wall is accompanied by the development of vasa vasorum, to meet the need for additional supply of nutrients and oxygen. Endothelial dysfunction of vasa vasorum contributes to the development of atherosclerosis. Atherosclerotic lesions are predominant in the intima, although atherosclerosis can affect all three layers of the vascular wall. The characteristic feature of atherosclerotic lesions is the accumulation of lipoproteins in the subintimal layer, migration of inflammatory cells to the intima, and loss of media immune privilege. The VSMCs in the intima can transform from the contractile phenotype to synthetic, chondrocyte, osteocyte, adipocyte, or macrophage-foam cell type, and the fibroblasts transform into myofibroblasts. PVAT also contributes to the proatherogenic state, by activating immune cells in ATLOs to secrete proinflammatory and proatherogenic cytokines.

Since each cell type contributes to the development and progression of atherosclerosis, each cell type represents a potential therapeutic target. By modulating these cells to change their function from proatherogenic to antiatherogenic, we may be able to alter, slow down, or mitigate the course of atherosclerosis. In addition, vasa vasorum may be a particularly important target in the treatment of atherosclerosis, as atherosclerosis usually affects the segments of vessel wall supplied by vasa vasorum.
